# Clinical outcome after undisplaced femoral neck fractures: A prospective comparison of 14,757 undisplaced and displaced fractures reported to the Norwegian Hip Fracture Register

**DOI:** 10.3109/17453674.2011.588857

**Published:** 2011-07-08

**Authors:** Jan-Erik Gjertsen, Jonas M Fevang, Kjell Matre, Tarjei Vinje, Lars B Engesæter

**Affiliations:** ^1^The Norwegian Arthroplasty Register, Department of Orthopaedic Surgery, Haukeland University Hospital; ^2^Department of Surgical Sciences, University of Bergen, Bergen, Norway

## Abstract

**Background and purpose:**

Little attention has been paid to undisplaced femoral neck fractures. By using data from the Norwegian Hip Fracture Register, we investigated the risk of reoperation and the clinical outcome after treatment of these fractures in patients over 60 years of age.

**Methods:**

Data on 4,468 patients with undisplaced femoral neck fractures who were operated with screw osteosynthesis were compared to those from 10,289 patients with displaced femoral neck fractures treated with screw osteosynthesis (n = 3,389) or bipolar hemiarthroplasty (n = 6,900). The evaluation was based on number of reoperations and patient assessment at 4 and 12 months of follow-up.

**Results:**

The 1-year implant survival was 89% after screw fixation for undisplaced fractures, 79% after screw fixation for displaced fractures, and 97% after hemiarthroplasty for displaced fractures. Patients with displaced fractures who were operated with internal fixation had a higher risk of reoperation (RR = 1.9, CI: 1.7–2.2), reported more pain, were less satisfied, and had lower quality of life than patients with undisplaced fractures treated with internal fixation (p < 0.05). Patients with displaced fractures who were operated with hemiarthroplasty had a lower risk of reoperation than patients with undisplaced fractures who were operated with internal fixation (RR = 0.32, CI: 0.27–0.38). Furthermore, they had the lowest degree of pain, were most satisfied, and reported the highest quality of life.

**Interpretation:**

**Interpretation** The differences in clinical outcome found were less than what is considered to be of clinical importance. The results support the use of screw osteosynthesis for undisplaced femoral neck fractures in elderly patients, although even better results were obtained in the hemiarthroplasty group in patients with displaced fractures.

Each year, approximately 9,000 patients are operated for hip fractures in Norway. 19% are undisplaced femoral neck fractures (Garden 1 and 2) and 38% are displaced (Gjertsen et al. 2008). The treatment of displaced fractures has been investigated extensively, and some recent reports have shown better clinical outcome after hemiarthroplasty than after screw fixation (Rogmark et al. 2002, Frihagen et al. 2007, Gjertsen et al. 2010). Less has been published on the treatment of undisplaced fractures. Some authors advocate internal screw fixation as being the best treatment, even though a high rate of reoperations has been reported (Conn and Parker 2004, Bjorgul and Reikeras 2007, Parker et al. 2008). A recent study found poor outcome in many patients after treatment of undisplaced fractures (Rogmark et al. 2009).

In Norway, there are no national guidelines for the treatment of hip fractures. The standard treatment for undisplaced fractures has been internal fixation with 2 screws or pins (94% of fractures) (Gjertsen et al. 2008). In the present study, we wanted to investigate the results after undisplaced femoral neck fractures, as reported to the Norwegian Hip Fracture Register. Risk of reoperation, pain, patient satisfaction, and quality of life were used as outcome measures. Since the undisplaced fractures were almost exclusively treated with internal fixation, patients with displaced femoral neck fractures treated with screw osteosynthesis or hemiarthroplasty were used as reference groups when analyzing the results.

## Patients and methods

Since January 1, 2005, the Norwegian Hip Fracture Register (NHFR) has recorded fractures of the proximal femur as a prospective observational study (Gjertsen et al. 2008). The completeness of primary operations in the NHFR (when compared to the Norwegian Patient Registry) was 79% after 2 years of registration (Gjertsen et al. 2008). The number of reported hip fractures has, however, increased in recent years, and the degree of completeness is probably higher today (Engesaeter et al. 2010). No completeness studies of reoperations in the NHFR have been done. Reoperations involving total hip arthroplasties are reported to the Norwegian Arthroplasty Register (NAR) with a completeness of over 98% (Espehaug et al. 2006). These particular reoperations had been added to the NHFR database before analyses were performed. For the NHFR, after each operation, patient data and operative data are filled in by the surgeon on a standard one-page form and sent to the registry. Both primary operations and reoperations are registered, and reoperations are linked to the index operation using the national identification number assigned to each inhabitant of Norway.

The definition of a reoperation is any operation performed due to complications after the index operation. In the present study, only reoperations during the first year postoperatively were analyzed. Although each patient could have more than one reoperation, only the first one was counted in the study. Based on the patient's medical record, or with the help of relatives, the cognitive function should be defined for all patients. If in doubt about the patient's cognitive function, the surgeons contributing to the registry were recommended to use the clock-drawing test (Shulman 2000).

All patients received a questionnaire from the register 4 and 12 months after the primary operation. This questionnaire included a visual analog scale (VAS) for average pain from the operated hip during the previous month (0 = no pain, 100 = worst pain) and a VAS for satisfaction with the result of the operation (0 = very satisfied, 100 = very unsatisfied). Furthermore, the questionnaire included the Norwegian translation of the EuroQol, which is a non-disease-specific instrument for describing and evaluating health-related quality of life (EQ-5D) (Brooks 1996). The EQ-5D index scores generated from a large European population were used (Greiner et al. 2003). An EQ-5D index score of 1 represents the best possible health state, and a score of 0 represents a health state similar to death. The preoperative EQ-5D was filled in retrospectively, 4 months after surgery.

**Figure 1.**
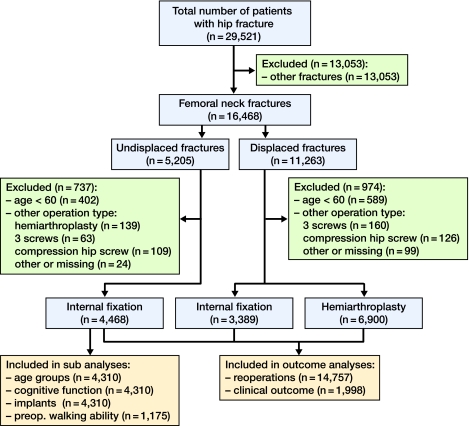
Flow chart of patients.

As of April 14, 2009, there were 29,521 primary operations due to hip fractures registered in the NHFR for the period 2005–2008 (Figure 1). Of these fractures, 16,468 were femoral neck fractures. 94% of the undisplaced femoral neck fractures (Garden 1 and 2) were operated with 2 screws/pins. Displaced femoral neck fractures (Garden 3 and 4) were operated in most cases (95%) with either 2 screws/pins or a bipolar hemiarthroplasty. Fractures operated with other methods were excluded from further analysis. In order to obtain comparable treatment groups, patients younger than 60 years of age were excluded. Finally, 14,757 patients were included in the outcome analysis comparing reoperation rate in 3 treatment groups (undisplaced fracture with screw osteosynthesis: n = 4,468; displaced fracture with screw osteosynthesis: n = 3,389; and displaced fracture with hemiarthroplasty: n = 6,900) (Figure 1). In the analysis of clinical outcome, only patients with complete 4- and 12-month questionnaires were included (n = 1,998).

To investigate possible risk factors for reoperation after undisplaced femoral neck fractures, subanalyses were performed with patients divided into different age groups (60–69, 70–79, 80–89, and > 90 years) and groups according to their cognitive function (no dysfunction, dysfunction, and uncertain). Furthermore, we performed subanalyses on patients with undisplaced fractures that were operated with different types of screws, and analyses on patients with different preoperative walking ability using the 3 mobility levels in the EQ-5D questionnaire (no problems in walking, some problems in walking, confined to bed).

All patients remained in their initial treatment groups according to the intention-to-treat-principle. The Norwegian Data Inspectorate approved the recording of data.

### Statistics

The Pearson chi-square test was used for comparison of categorical variables. Analysis of variance (ANOVA) was used for continuous variables. The analyses of preoperative EQ-5D index scores presented in Table 1 and the patient-assessed outcomes presented in Table 3 were adjusted for potential confounders (age, sex, cognitive function, and comorbidity (ASA-classification)) using general linear models (GLMs). In the reoperation analyses, a Cox regression model was used to adjust for differences in age (age groups), sex, cognitive function, and comorbidity (ASA classification). Furthermore, in the subanalyses of undisplaced fractures, adjustments for type of implant were also done. We used the Cox regression model to calculate differences in revision risk for the different treatment groups. In order to get the same observational time for both clinical outcome and reoperations, only reoperations performed during the first year postoperatively were included in this study. Consequently, all patients were followed until time of revision or they were censored 1 year postoperatively or at time of death, which was obtained from Statistics Norway. The proportional hazards assumption was not fulfilled when investigated visually by use of log-minus-log plots. Relative risks and all continuous variables are presented with 95% confidence intervals (CIs). We did not adjust for patients who were operated on both sides. All p-values were 2-tailed, and the significance level was set at 0.05. The analyses were performed using SPSS software version 17.0.

**Table 1.** Baseline characteristics of patients

	Undisplaced fracture	Displaced fracture	Displaced fracture	p-value
	Internal fixation	Internal fixation	Hemiarthroplasty	
Total number	4,468	3,389	6,900	
Mean age [SD], years (95% CI)	81 [8.4]	81 [8.9]	83 (7.0)	< 0.001**^a^**
	(81–81)	(81–81)	(83–83)	
Women (%)	3,111 (80)	2,308 (68)	5,221 (76)	< 0.001 **^b^**
ASA class (%)				
ASA 1	457 (10)	301 (8.9)	364 (5.3)	
ASA 2	1,705 (38)	1,126 (33)	2,418 (35)	
ASA 3	2,013 (45)	1,610 (48)	3,613 (52)	
ASA 4	219 (4.9)	296 (8.7)	387 (5.6)	
ASA 5	3 (0.1)	14 (0.4)	3 (0.1)	
Missing	71 (1.6)	42 (1.2)	115 (1.7)	< 0.001 **^b^**
Cognitive impairment (%)	1,082 (24)	941 (28)	1,741 (25)	0.002 **^b^**
Preop. EQ-5D score [SD] (95% CI)	0.64 [0.03]	0.62 [0.03]	0.64 [0.03]	0.2 **^c^**
	(0.59–0.70)	(0.57–0.68)	(0.58–0.69)	

**^a^** ANOVA.

**^b^** Pearson chi-square test.

**^c^** GLM with adjustment for age, sex, comorbidity (ASA classification), and cognitive function.

**Table 3.** Type of reoperation

Type of reoperation	Undisplaced fracture	Displaced fracture	Displaced fracture
	Internal fixation	Internal fixation	Hemiarthroplasty
	n (%) ^a^	n (%) ^a^	n (%) ^a^
Total hip arthroplasty**^b^**	108 (25)	150 (26)	10 (4.7)
Bipolar hemiarthroplasty	263 (60)	337 (57)	14 (6.6)
Unipolar hemiarthroplasty	2 (0.5)	16 (2.7)	
Removal of implant	15 (3.4)	44 (7.5)	1 (0.5)
Re-osteosynthesis	27 (6.2)	17 (2.9)	
Girdlestone procedure	10 (2.3)	21 (3.6)	16 (7.5)
Drainage of hematoma or infection	11 (2.5)	2 (0.3)	93 (44)**^c^**
Open reduction of dislocated hemiarthroplasty			26 (12)**^d^**
Closed reduction of dislocated hemiarthroplasty			12 (5.7)
Other		1 (0.2)	40 (19)
Total no. of reoperations	436 (9.8)**^e^**	588 (17)**^e^**	212 (3.1)**^e^**

**^a^** Percentage of reoperated hips.

**^b^** Reported to the Norwegian Arthroplasty Register.

**^c^** Procedure included change of bipolar head in 48 patients.

**^d^** Procedure included change of bipolar head in 14 patients.

**^e^** Percentage of primary-operated hips.

## Results

The patients with displaced fractures operated with hemiarthroplasty were older, were more often female, and had more comorbidity than the patients operated with internal fixation. The patients with displaced fractures operated with internal fixation were more often cognitively impaired than patients in the other two treatment groups. There was no statistically significant difference in preoperative EQ-5D index score between the different treatment groups (Table 1).

In the patients treated with screw osteosynthesis, the most frequently used implants were Olmed screws (DePuy) (58%) and the Richards CHP (Smith and Nephew) (25%). In the hemiarthroplasty group, 81% of the prostheses were cemented (n = 5,612) (Table 2; see supplementary data).

### Reoperations

After 1 year of follow-up, implant survival (the percentage of patients who were not reoperated) was 89% for undisplaced fractures operated with internal fixation, 79% for displaced fractures operated with internal fixation, and 97% for displaced fractures operated with hemiarthroplasty (Kaplan-Meier) (Table 3).

**Figure 2.**
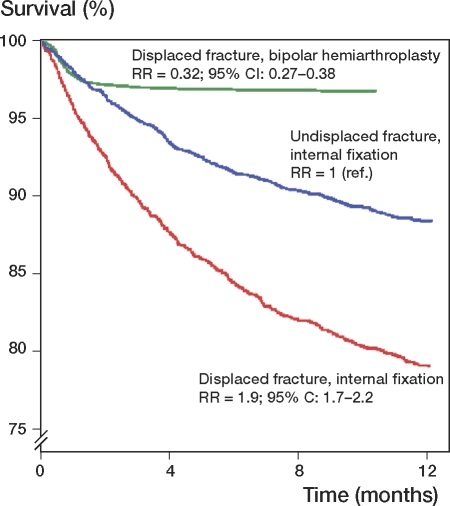
Adjusted survival of implants for the different treatment groups (n = 14,757).

After adjustments for differences in age, sex, cognitive impairment, and comorbidity, the displaced fractures operated with internal fixation had a higher risk of reoperation than the undisplaced fractures operated with internal fixation (RR = 1.9, CI: 1.7–2.2; p < 0.001). The displaced fractures operated with hemiarthroplasty had a lower risk of reoperation than the undisplaced fractures operated with internal fixation (RR = 0.32, CI: 0.27–0.38; p < 0.001) (Figure 2).

### Clinical outcome

1,998 patients completed both patient questionnaires and were included in the analyses of clinical outcome (Figure 1). Excluding patients who died within the first year postoperatively, the response rates for patients treated with screw osteosynthesis for undisplaced and displaced fractures were 19% (670/3,590) and 22% (550/2,555) respectively. For patients treated with hemiarthroplasty for displaced fractures, the response rate was 15% (778/5,317).

Patients operated with hemiarthroplasty due to displaced fractures reported higher satisfaction with the result of the operation, less pain, and a higher quality of life (EQ-5D index score) at both the 4-month and the 12-month follow-up, compared to patients with undisplaced fractures operated with internal fixation. Patients with displaced fractures operated with internal fixation reported lower satisfaction with the result of the operation, more pain, and a lower quality of life (EQ-5D index score)—at both four and twelve months—compared to patients with undisplaced fractures operated with internal fixation (Table 4).

**Table 4.** Comparison of patient-assessed outcomes in patients according to type of fracture and treatment. Intention-to-treat-analysis. Values are mean (95% CI) [SD]

	Undisplaced fracture	Displaced fracture	p-value **^a^**	Displaced fracture	p-value **^a^**
	Internal fixation	Internal fixation	GLM	Hemiarthroplasty	GLM
	(n = 670)	(n = 550)		(n = 778)	
Patient satisfaction **^b^**					
At 4 months	26 (19–34) [4.1]	34 (26–42) [4.1]	< 0.001	20 (12–28) [4.1]	< 0.001
At 12 months	29 (20–37) [4.2]	35 (27–44) [4.2]	< 0.001	23 (15–31) [4.2]	< 0.001
Pain **^c^**					
At 4 months	27 (19–35) [3.9]	33 (25–40) [3.8]	< 0.001	19 (12–27) [3.9]	< 0.001
At 12 months	26 (19–34) [3.8]	30 (22–37) [3.8]	0.007	19 (12–26) [3.8]	< 0.001
EQ-5D index score**^d^**					
At 4 months (n = 303)	0.50 (0.41–0.59) [(0.04]	0.44 (0.35–0.52) [0.04]	< 0.001	0.53 (0.44–0.62) [0.05]	0.02
At 12 months (n = 292)	0.60 (0.50–0.69) [0.05]	0.55 (0.46–0.64) [0.05]	0.003	0.63 (0.54–0.72) [0.05]	0.02

**^a^** p-value is the probability of no difference between displaced and undisplaced fractures (general linear models (GLMs) adjusted for differences in age, sex, cognitive impairment, and ASA class between the groups).

**^b^** Patient satisfaction: VAS (visual analog scale); 0 = satisfied and 100 = dissatisfied.

**^c^** Pain: VAS; 0 = no pain and 100 = unbearable pain.

**^d^** EQ-5D index score: 0 = the worst possible health state and 1.0 = full health. EQ-VAS.

### Subanalysis of undisplaced fractures

**Figure 3.**
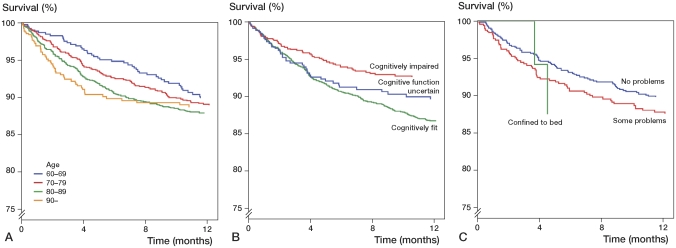
Adjusted survival of implants: A. for undisplaced fracture according to age group (n = 4,310); B. for undisplaced fracture according to cognitive function (n = 4,310); C. for undisplaced fracture according to preoperative walking ability (n = 1,175).

4,310 patients were included in the subanalyses of undisplaced fractures (Figure 1). We found no effect on the risk of reoperation of age group, sex, and ASA classification in a Cox regression analysis (Table 5; see supplementary data). There was a trend towards fewer reoperations in the youngest patients, but no statistically significant differences were found (Figure 3A). Patients with cognitive impairment had a reduced risk of reoperation compared to the cognitively fit patients (RR = 0.57, CI: 0.44–0.75; p < 0.001) (Figure 3B). Furthermore, type of implant had an influence on the risk of reoperation. Compared to Olmed screws (DePuy), the Asnis III screws (Stryker) had almost double the risk of reoperation (RR = 2.1, CI: 1.5–3.0; p < 0.001). The 34 reoperations performed after osteosynthesis with Asnis III screws included 32 major procedures (4 total hip arthroplasties and 28 hemiarthroplasties) and 2 minor procedures (removal of screws).

We also conducted a subanalysis on patients with undisplaced fractures where there was information on preoperative walking ability (n = 1,175). 11% of the patients with no walking problems prior to the fracture were reoperated (74/687) and 13% of the patients with some walking problems were reoperated (61/471), whereas 2 of 17 patients who had been confined to bed preoperatively were reoperated. Cox regression analysis with adjustment for differences in age group, sex, cognitive function, comorbidity (ASA classification), and type of implant showed that preoperative walking ability had no influence on the reoperation rate (p = 0.5) (Figure 3C).

## Discussion

Our main findings were that screw fixation for undisplaced femoral neck fractures led to fewer reoperations, more satisfied patients, less pain, and higher quality of life compared to screw fixation for displaced fractures. However, the clinical outcome was inferior to that reported by patients treated with a bipolar hemiarthroplasty (HA) for displaced femoral neck fractures. In patients treated with internal fixation (IF) after undisplaced femoral neck fracture, there were fewer reoperations performed on cognitively impaired patients than on cognitively lucid patients.

### Reoperations

Not surprisingly, the reoperation rate for the undisplaced fractures was relatively low compared to that for the displaced fractures treated with IF. On the other hand, more reoperations were done after treatment of undisplaced femoral neck fractures with IF than after treatment of displaced fractures with a bipolar HA. The reoperation rate for the undisplaced fractures of 11% is lower than in other studies, which found reoperation rates of between 13% and 19% (Conn and Parker 2004, Bjorgul and Reikeras 2007, Parker et al. 2008, Rogmark et al. 2009). Also, the reoperation rate for HA, particularly the reoperation rate due to dislocations, was low in our study. This might be explained by an incomplete reporting of minor reoperations to the register (i.e. closed reduction of dislocated hemiprostheses or removal of osteosynthesis material). Most reoperations reported after IF for undisplaced fractures were major procedures which may, at least temporarily, have reduced the quality of life of these patients.

One important limitation of our study is the short follow-up. Complications after internal fixation—such as sequelae from avascular necrosis of the femoral head, non-union, or malunion—giving symptoms that develop later than 12 months postoperatively will not have been registered in our study. For the HAs, the possibility of detecting femoral loosening and acetabular erosion increases with longer follow-up (Soreide et al. 1982, Baker et al. 2006). Leonardsson et al. (2010) reported a reoperation rate of 8% after HA for displaced fractures with 10 years of follow-up. The long-term results of HAs are still uncertain, however, and must be addressed in future studies.

The definition of cognitive impairment in the hip fracture register is rather crude. Still, the subanalyses reflect a difference between patients classified as cognitively impaired and patients classified as cognitively lucid. In the Cox regression analyses, we found that there were fewer reoperations after undisplaced fractures in cognitively impaired patients. One explanation could be that these patients may have had difficulties in expressing their problems and pain, and were less frequently checked and examined postoperatively. The inferior results of HAs in cognitively impaired patients that have been reported previously in some studies (van Dortmont et al. 2000, Blomfeldt et al. 2005) and concern about the increased risk of dislocation reported after total hip replacement in cognitively impaired patients (Johansson et al. 2000) may result in some reluctance among the surgeons to perform further surgery after complications in these patients. However, more recent studies have found good results also in cognitively impaired patients when modern HAs were used (Frihagen et al. 2007, Gjertsen et al. 2010). Thus, to a greater degree than others, these patients could be primarily treated with an HA.

The Asnis III screws gave double the risk of reoperation compared to Olmed screws, but they were only used at 5 hospitals, and in a few patients. Consequently, the results must be interpreted with caution as the differences may be explained by factors such as better clinical follow-up of the patients and a lower threshold for performing reoperations at these specific hospitals.

### Clinical outcome

Most differences in clinical outcome (pain, patient satisfaction, and quality of life) in our study reached statistical significance. Because of the large number of patients, we must also consider what is clinically significant. Based on earlier studies on clinically significant differences using visual analog scales (Ehrich et al. 2000) and EQ-5D index score (Walters and Brazier 2005, Pickard et al. 2007), a difference of 10 on the VAS and a difference of 0.07 on the EQ-5D index score may indicate a clinically significant difference. Thus, none of the differences found between undisplaced and displaced fractures reached a clinically significant level. However, there was a trend indicating better results for displaced femoral neck fractures treated with HA than for undisplaced fractures treated with IF. One limitation of our study was the low response rates to the patient questionnaires (15–22%). As clinical outcome may influence the response rate, and as there were different response rates between the groups, this could mean that bias was introduced into our results.

The differences found between the undisplaced and displaced femoral neck fractures treated with IF justify the use of the Garden classification as a useful tool when treating these fractures. We found that a considerable number of major reoperations were performed after internal fixation for undisplaced fractures. Furthermore, there was a trend towards better clinical outcome after HA for displaced femoral neck fractures compared to IF for undisplaced fractures. If we assume that the superior results of HA for displaced fractures could also be found for undisplaced fractures, this could indicate that undisplaced femoral neck fractures could also be treated primarily with HA. On the other hand, the results obtained with IF for undisplaced fractures were better than for displaced fractures, making this treatment method more acceptable in this fracture group. A randomized, controlled trial (RCT) comparing the results of IF and HA for treatment of undisplaced femoral neck fractures would give a final answer to this question, although it might be difficult to do: the follow-up must be long enough to allow evaluation of long-term complications as well. Due to high mortality rates in these patients, an RCT would probably require up to 350 patients in each group in order to be able to detect clinically significant differences in functional outcome, and differences in reoperation rates similar to those found in our study. Considering the better clinical results for operation with bipolar HA, the mortality rates for the two treatment groups should also be compared. However, this is probably best done with a cohort study, as such a study would require several thousand patients.

## Supplementary data

Tables 2 and 5 are available at our website (www.actaorthop.org), identification number 4442.

## References

[CIT0001] Baker RP, Squires B, Gargan MF, Bannister GC (2006). Total hip arthroplasty and hemiarthroplasty in mobile, independent patients with a displaced intracapsular fracture of the femoral neck. A randomized, controlled trial. J Bone Joint Surg (Am).

[CIT0002] Bjorgul K, Reikeras O (2007). Outcome of undisplaced and moderately displaced femoral neck fractures. Acta Orthop.

[CIT0003] Blomfeldt R, Tornkvist H, Ponzer S, Soderqvist A, Tidermark J (2005). Internal fixation versus hemiarthroplasty for displaced fractures of the femoral neck in elderly patients with severe cognitive impairment. J Bone Joint Surg (Br).

[CIT0004] Brooks R (1996). EuroQol: the current state of play. Health Policy.

[CIT0005] Conn KS, Parker MJ (2004). Undisplaced intracapsular hip fractures: results of internal fixation in 375 patients. Clin Orthop.

[CIT0006] Ehrich EW, Davies GM, Watson DJ, Bolognese JA, Seidenberg BC, Bellamy N (2000). Minimal perceptible clinical improvement with the Western Ontario and McMaster Universities osteoarthritis index questionnaire and global assessments in patients with osteoarthritis. J Rheumatol.

[CIT0007] Engesaeter L, Furnes O, Havelin LI, Fenstad AM (2010). The Norwegian Arthroplasty Register. Annual report.

[CIT0008] Espehaug B, Furnes O, Havelin LI, Engesaeter LB, Vollset SE, Kindseth O (2006). Registration completeness in the Norwegian Arthroplasty Register. Acta Orthop.

[CIT0009] Frihagen F, Nordsletten L, Madsen JE (2007). Hemiarthroplasty or internal fixation for intracapsular displaced femoral neck fractures: randomised controlled trial. BMJ.

[CIT0010] Gjertsen JE, Engesaeter LB, Furnes O, Havelin LI, Steindal K, Vinje T, Fevang JM (2008). The Norwegian Hip Fracture Register. Experiences after the first 2 years and 15,576 reported hips. Acta Orthop.

[CIT0011] Gjertsen JE, Vinje T, Engesaeter LB, Lie SA, Havelin LI, Furnes O, Fevang JM (2010). Internal Screw Fixation Compared with Bipolar Hemiarthroplasty for Treatment of Displaced Femoral Neck Fractures in Elderly Patients. J Bone Joint Surg (Am).

[CIT0012] Greiner W, Weijnen T, Nieuwenhuizen M, Oppe S, Badia X, Busschbach J, Buxton M, Dolan P, Kind P, Krabbe P, Ohinmaa A, Parkin D, Roset M, Sintonen H, Tsuchiya A, de Charro F (2003). A single European currency for EQ-5D health states. Results from a six-country study. Eur J Health Econ.

[CIT0013] Johansson T, Jacobsson SA, Ivarsson I, Knutsson A, Wahlstrom O (2000). Internal fixation versus total hip arthroplasty in the treatment of displaced femoral neck fractures: a prospective randomized study of 100 hips. Acta Orthop Scand.

[CIT0014] Leonardsson O, Sernbo I, Carlsson A, Akesson K, Rogmark C (2010). Long-term follow-up of replacement compared with internal fixation for displaced femoral neck fractures: results at ten years in a randomised study of 450 patients. J Bone Joint Surg (Br).

[CIT0015] Parker MJ, White A, Boyle A (2008). Fixation versus hemiarthroplasty for undisplaced intracapsular hip fractures. Injury.

[CIT0016] Pickard AS, Neary MP, Cella D (2007). Estimation of minimally important differences in EQ-5D utility and VAS scores in cancer. Health Qual Life Outcomes.

[CIT0017] Rogmark C, Carlsson A, Johnell O, Sernbo I (2002). A prospective randomised trial of internal fixation versus arthroplasty for displaced fractures of the neck of the femur. Clinical outcome for 450 patients at two years. J Bone Joint Surg (Br).

[CIT0018] Rogmark C, Flensburg L, Fredin H (2009). Undisplaced femoral neck fractures--no problems? A consecutive study of 224 patients treated with internal fixation. Injury.

[CIT0019] Shulman KI (2000). Clock-drawing: is it the ideal cognitive screening test?. Int J Geriatr Psychiatry.

[CIT0020] Soreide O, Skjaerven R, Alho A (1982). The risk of acetabular protrusion following prosthetic replacement of the femoral head. Acta Orthop Scand.

[CIT0021] van Dortmont LM, Douw CM, van Breukelen AM, Laurens DR, Mulder PG, Wereldsma JC, van Vugt AB (2000). Cannulated screws versus hemiarthroplasty for displaced intracapsular femoral neck fractures in demented patients. Ann Chir Gynaecol.

[CIT0022] Walters SJ, Brazier JE (2005). Comparison of the minimally important difference for two health state utility measures: EQ-5D and SF-6D. Qual Life Res.

