# Supplementary material for: Clinical outcome after undisplaced femoral neck fractures: A prospective comparison of 14,757 undisplaced and displaced fractures reported to the Norwegian Hip Fracture Register
Source: Acta Orthop. 2011 Jul 8;82(3):268–74. doi: 10.3109/17453674.2011.588857 (PMC3235303; doi:10.3109/17453674.2011.588857)
Supplement: Supplementary file 1 [file ORT-1745-3674-82-268-s4442.pdf]

Supplementary article data

Clinical outcome after undisplaced femoral neck fractures

A prospective comparison of 14,757 undisplaced and displaced fractures reported to the Norwegian Hip Fracture Register

Jan-Erik Gjertsen[1], Jonas M Fevang[1], Kjell Matre[1], Tarjei Vinje[1], and Lars B Engesæter[1,2]

[1]The Norwegian Arthroplasty Register, Department of Orthopaedic Surgery, Haukeland University Hospital; [2]Department of Surgical Sciences, University of Bergen, Bergen, Norway
Correspondence: jan-erik.gjertsen@helse-bergen.no
Submitted 10-10-20. Accepted 11-02-22

Table 2. Types of implants

Implant	n (%)	n (%)
Undisplaced fracture, internal fixation	4,468 (100)	
Olmed (DePuy)		2,601 (58)
Richards CHP (Smith and Nephew)		1,130 (25)
Hansson Pin System (Swemac)		524 (12)
Asnis III (Stryker)		212 (4.7)
Missing		1
Displaced fracture, internal fixation	3,389 (100)	
Olmed (DePuy)		2,030 (60)
Richards CHP (Smith and Nephew)		694 (20)
Hansson Pin System (Swemac)		522 (15)
Asnis III (Stryker)		142 (4.2)
Missing		1
Displaced fracture, hemiarthroplasty	6,900 (100)	
Exeter – UHR (Stryker)		1,667 (24)
Charnley – Hastings (DePuy)		1,593 (23)
Corail – Landos Bipolar Cup (DePuy)		975 (14)
Titan – Landos Bipolar Cup (DePuy)		723 (10)
Spectron – TANDEM (Smith and Nephew)		568 (8.2)
Charnley Modular (DePuy)		388 (5.6)
Lubinus SPII-Vario-cup (LINK)		330 (4.2)
Other/missing		656 (9.5)

Open Access - This article is distributed under the terms of the Creative Commons Attribution Noncommercial License which permits any noncommercial use, distribution, and reproduction in any medium, provided the source is credited.
DOI 10.3109/17453674.2011.588857

Table 5. Number of reoperations according to different patient characteristics and type of implant for patients with undisplaced fractures. Risk ratio (RR) and p-value were adjusted for differences in age group, sex, cognitive function, comorbidity (ASA classification), and type of implant in a Cox model

	No. of operations	No. of reoperations (%) [a]	Risk ratio	95% CI	p-value [c]
Total number	4,310 [b]	420 (9.7)			
Age group					0.3
60–69	520	47 (9.0)	1		
70–79	1,157	117 (10)	1.1	0.81–1.6	0.4
80–89	2,037	202 (9.9)	1.3	0.94–1.8	0.1
> 90	596	54 (9.1)	1.3	0.88–2.0	0.2
Sex					0.9
Male	1,307	129 (9.9)	1		
Female	3,003	291 (9.7)	0.88	0.71–1.1	0.9
Cognitive dysfunction					< 0.001
No	2,789	313 (11)	1		
Yes	1,063	66 (6.2)	0.57	0.44–0.75	< 0.001
Uncertain/missing	458	41 (9.0)	0.80	0.58–1.1	0.2
ASA class					0.3
ASA 1	455	40 (8.8)	1		
ASA 2	1,678	184 (11)	1.4	0.91–1.6	0.09
ASA 3	1,961	184 (9.4)	1.4	0.86–1.6	0.1
ASA 4	213	12 (5.6)	0.92	0.47–1.5	0.9
ASA 5	3	0 (0)			
Type of implant					< 0.001
Olmed (DePuy)	2,509	213 (8.5)	1		
Richards CHP (Smith and Nephew)	1,093	118 (11)	1.3	1.0–1.6	0.04
Hansson Pin System (Swemac)	506	55 (11)	1.3	0.95–1.7	0.1
Asnis III (Stryker)	201	34 (17)	2.1	1.5–3.0	< 0.001

[a] Percentage reoperated hips of total primary-operated hips.
[b] 158 patients with incomplete information on cognitive function or ASA classification were excluded from the analysis.
[c] Cox regression analysis.
